# Fenfluramine increases survival and reduces markers of neurodegeneration in a mouse model of Dravet syndrome

**DOI:** 10.1002/epi4.12873

**Published:** 2023-12-22

**Authors:** John Cha, Gregory Filatov, Steven J. Smith, Arnold R. Gammaitoni, Amélie Lothe, Thadd Reeder

**Affiliations:** ^1^ University of California San Francisco San Francisco California USA; ^2^ Zogenix, Inc. (now a part of UCB) Emeryville California USA; ^3^ Crosshair Therapeutics, Inc. Sunnyvale California USA; ^4^ WuXi AppTec, Inc. San Francisco California USA; ^5^ UCB Pharma S.A. Colombes France

**Keywords:** Dravet syndrome, fenfluramine, myelin, neuroinflammation, survival

## Abstract

**Objective:**

In patients with Dravet syndrome (DS), fenfluramine reduced convulsive seizure frequency and provided clinical benefit in nonseizure endpoints (e.g., executive function, survival). In zebrafish mutant *scn1* DS models, chronic fenfluramine treatment preserved neuronal cytoarchitecture prior to seizure onset and prevented gliosis; here, we extend these findings to a mammalian model of DS (*Scn1a* ^*+/−*^ mice) by evaluating the effects of fenfluramine on neuroinflammation (degenerated myelin, activated microglia) and survival.

**Methods:**

*Scn1a* ^*+/−*^ DS mice were treated subcutaneously once daily with fenfluramine (15 mg/kg) or vehicle from postnatal day (PND) 7 until 35‐37. Sagittal brain sections were processed for immunohistochemistry using antibodies to degraded myelin basic protein (D‐MBP) for degenerated myelin, or CD11b for activated (inflammatory) microglia; sections were scored semi‐quantitatively. Apoptotic nuclei were quantified by TUNEL assay. Statistical significance was evaluated by 1‐way ANOVA with post‐hoc Dunnett's test (D‐MBP, CD11b, and TUNEL) or Logrank Mantel‐Cox (survival).

**Results:**

Quantitation of D‐MBP immunostaining per 0.1 mm^2^ unit area of the parietal cortex and hippocampus CA3 yielded significantly higher spheroidal and punctate myelin debris counts in vehicle‐treated DS mice than in wild‐type mice. Fenfluramine treatment in DS mice significantly reduced these counts. Activated CD11b + microglia were more abundant in DS mouse corpus callosum and hippocampus than in wild‐type controls. Fenfluramine treatment of DS mice resulted in significantly fewer activated CD11b + microglia than vehicle‐treated DS mice in these brain regions. TUNEL staining in corpus callosum was increased in DS mice relative to wild‐type controls. Fenfluramine treatment in DS mice lowered TUNEL staining relative to vehicle‐treated DS mice. By PND 35‐37, 55% of control DS mice had died, compared with 24% of DS mice receiving fenfluramine treatment (*P* = 0.0291).

**Significance:**

This is the first report of anti‐neuroinflammation and pro‐survival after fenfluramine treatment in a mammalian DS model. These results corroborate prior data in humans and animal models and suggest important pharmacological activities for fenfluramine beyond seizure reduction.

**Plain Language Summary:**

Dravet syndrome is a severe epilepsy disorder that impairs learning and causes premature death. Clinical studies in patients with Dravet syndrome show that fenfluramine reduces convulsive seizures. Additional studies suggest that fenfluramine may have benefits beyond seizures, including promoting survival and improving control over emotions and behavior. Our study is the first to use a Dravet mouse model to investigate nonseizure outcomes of fenfluramine. Results showed that fenfluramine treatment of Dravet mice reduced neuroinflammation significantly more than saline treatment. Fenfluramine‐treated Dravet mice also lived longer than saline‐treated mice. These results support clinical observations that fenfluramine may have benefits beyond seizures.

## Abstract 2

Dravet syndrome is a severe epilepsy disorder that impairs learning and causes premature death. Clinical studies in patients with Dravet syndrome show that fenfluramine reduces convulsive seizures. Additional studies suggest that fenfluramine may have benefits beyond seizures, including promoting survival and improving control over emotions and behavior. Our study is the first to use a Dravet mouse model to investigate nonseizure outcomes of fenfluramine. Results showed that fenfluramine treatment of Dravet mice reduced neuroinflammation significantly more than saline treatment. Fenfluramine‐treated Dravet mice also lived longer than saline‐treated mice. These results support clinical observations that fenfluramine may have benefits beyond seizures.

**Key Points**

In an *Scn1a* ^*+/−*^ mouse model of Dravet syndrome, fenfluramine treatment:

- Protected against myelin damage in the hippocampus CA3 and parietal cortex.
- Reduced microglial activation in hippocampal perforant pathway and corpus callosum (genu).
- Inhibited apoptosis in corpus callosum.
- Enhanced survival.

## 1 INTRODUCTION

Dravet syndrome (DS) is a rare, severe, treatment‐resistant developmental and epileptic encephalopathy.^1^, ^2^ More than 85% of patients with DS present with pathogenic variants in the *SCN1A* gene,^2^ which encodes the alpha‐1 subunit of the Na_v_1.1 neuronal voltage‐gated sodium channel.^3^, ^4^ Na_v_1.1 is enriched in inhibitory gamma‐aminobutyric acid (GABA)‐ergic interneurons of the hippocampus and cortex.^5^ In DS, Na_v_1.1 haploinsufficiency impairs inhibitory GABAergic action potentials in forebrain structures, and results in deficits in structure and function in the hippocampal network.^5^, ^6^ In addition to causing seizures, Na_v_1.1 haploinsufficiency causes nonseizure comorbidities as a direct consequence of the underlying pathology rather than—or in addition to—being caused only by neurological damage secondary to seizures.^7^

In patients with DS, the antiseizure medication fenfluramine has proven, clinical benefit in reducing convulsive seizure frequency^8^, ^9^, ^10^, ^11^ and alleviating nonseizure comorbidities, including survival^12^ and regulation of behavior, emotions, and cognition (i.e., executive function).^8^, ^13^ Additional preclinical evidence in nonseizure rodent models supports a role for fenfluramine in improving spatial learning and memory—important aspects of cognition.^14^, ^15^ In the *scn1* mutant zebrafish model of DS, aberrant neuronal cytostructural architecture with loss of GABAergic dendritic arborization occurred prior to seizure onset; in *scn1* mutants, fenfluramine treatment restored GABAergic dendritic arborization and gliosis to normal, wild‐type levels.^16^ Further, in the DBA/1 audiogenic seizure mouse model of sudden unexpected death in epilepsy (SUDEP), fenfluramine treatment reduced seizure‐induced respiratory arrest (S‐IRA) prior to SUDEP at a dose that did not significantly change seizure frequency compared to controls.^17^ Taken together, these results suggest that fenfluramine has additional, potentially disease‐modifying activities beyond seizure control.

The complex pharmacology of fenfluramine is likely responsible for affecting these seizure and nonseizure morbidities. Both in vitro and in vivo evidence supports fenfluramine acting as a potent serotonin releaser with agonist activity at the serotonin (5‐HT) receptor subtypes 5‐HT_2A_, 5‐HT_2C_, and 5‐HT_1D_.^18^, ^19^, ^20^ In vivo evidence supports agonist activity at the 5‐HT_4_ receptor^21^—a receptor associated with learning and memory in clinical studies.^22^ Additionally, fenfluramine positively modulates the activity of the Sigma1 receptor (Sigma1R), potentially in conjunction with endogenous neuro(active) steroids.^14^, ^15^

Mouse models of DS recapitulate features of the DS clinical phenotype, including nonseizure comorbidities such as alterations in emotions (e.g., anxiety) and deficits in behavior, cognition, and social interaction,^23^ as well as spontaneous seizures and increased mortality.^24^ These phenotypic changes are accompanied by astrogliosis, microgliosis, and altered neuronal morphology with impaired neurogenesis. Histopathology in the brains of DS mice includes aberrant myelination,^25^ neuroinflammation,^23^ and increased apoptosis.^26^ These findings support clinical evidence in DS, where white matter damage is evident on MRI^27^ and in resected brain sections at autopsy.^28^, ^29^ White matter damage is increasingly recognized as a common epileptogenic pathology.^30^, ^31^ Conditional knockout and knockdown studies in DS models suggest that nonseizure comorbidities may occur as primary consequences of the *Scn1a* mutation in addition to secondary consequences of seizure burden,^32^, ^33^, ^34^, ^35^, ^36^, ^37^, ^38^, ^39^, ^40^ confirming clinical reports.^7^

The clinical data provide incontrovertible evidence that fenfluramine suppresses convulsive seizures in patients with DS.^8^, ^9^, ^10^, ^11^ Evidence in clinical studies,^8^, ^13^ non‐mammalian DS models,^16^ and mammalian non‐DS models^17^ suggests that fenfluramine may also affect phenotypes that are caused by *Scn1a* haploinsufficiency but not necessarily secondary to seizures. Here, we present the first study to investigate the effect of fenfluramine on myelination, neuroinflammation, and apoptosis in the *Scn1a* ^*+/−*^ DS mouse brain, as well as survival as an endpoint in the *Scn1a* ^*+/−*^ DS mouse model.

## 2 METHODS

### 2.1 Study ethics

All experiments were approved by the Molecular Medicine Research Institute (MMRI; Sunnyvale, CA) Institutional Animal Care and Use Committee (IACUC) and conformed to the National Institutes of Health Guide for the Care and Use of Laboratory Animals.

### 2.2 Mice

The *Scn1a* ^*+/−*^ transgenic DS mouse model has been previously described by Kearney et al.^24^, ^41^ The heterozygous Dravet mice (*Scn1a* ^*tm1Kea*^ 50% C57BL/6J, 50% 129S6/SvEvTac background strain) were from The Jackson Laboratory (Bar Harbor, ME, USA). Briefly, a heterozygous *Scn1a* ^*+/−*^ null allele was generated in TL1 embryonic stem (ES) cells by targeted deletion of the first coding exon (129S6/SvEvTac). These mice were established and maintained as a co‐isogenic strain on the 129S6/SvEvTac (129) background. Crossing 129.*Scn1a* ^*+/−*^ mice to strain B6 resulted in (B6x129) F1.*Scn1a* ^*+/−*^ (F1.*Scn1a* ^*+/−*^) offspring.^24^ Genotype was confirmed at postnatal day (PND) 14 and again at harvest by real‐time PCR of tail tips at Transnetyx (Cordova, TN) with a custom primer‐probe set based upon those applied by Miller et al.^24^

Animals were housed in temperature‐controlled environments with a 12‐hour light/dark cycle and ad libitum access to food and water. Experiments used male and female mice in approximately equal ratios.

### 2.3 Treatment

Treatment was administered by subcutaneous injection to *Scn1a* ^*+/−*^ mice once daily from PND 7 through PND 35‐37. For comparison, wild‐type mice were injected with vehicle or fenfluramine in parallel with *Scn1a* ^*+/−*^ mice. An injection volume of 10 μL/g body weight was used to deliver dosages of 15 mg/kg racemic fenfluramine (Penn Pharma/PCI Pharma Services, Tredegar, Gwent, Wales; dissolved in saline) or 0.9% saline solution (vehicle control). PND ages for this study were selected to avoid periods of high demyelination and microglial activity associated with development; normal axonodendritic, axonosomatic, and axonoaxonic synaptic pruning occurs in all mice, with peak activity at PND 21.^42^, ^43^ Dose of fenfluramine was chosen based on the lowest reported dose to affect survival in the DBA/1 mouse model (15 mg/kg/day intraperitoneal).^17^ A dose of 15 mg/kg/day fenfluramine was based on calculations to determine the conversion factor necessary to account for interspecies differences in pharmacokinetics and metabolism (see references^17^, ^44^). In the DBA/1 mouse model, 15 mg/kg/day is pharmacokinetically equivalent to a 0.7 mg/kg/day dose of fenfluramine in humans, within the therapeutically effective dose range that Dravet patients are treated clinically (0.2‐0.7 mg/kg/day; 26 mg/day maximum).^8^, ^9^, ^10^, ^11^ Subcutaneous mode of administration was chosen due to the small size of the animals early in the experiment (closer to PND 7) and the associated risk in puncturing and damaging the viscera if using the intraperitoneal mode.

### 2.4 Tissue preparation

At the conclusion of the dosing period, surviving mice were deeply anesthetized with isoflurane and exsanguinated by cardiac puncture, then formalin‐fixed by whole body perfusion using 6 mL ice‐cold phosphate buffered saline, followed by 6 mL ice‐cold 4% paraformaldehyde. Right brains were harvested and processed as formalin‐fixed, paraffin embedded (FFPE) specimens by incubating in 10% neutral buffered formalin at room temperature for 24 hours, transferring to phosphate buffered saline containing 0.02% sodium azide preservative, and embedding in paraffin blocks. FFPE specimens were sectioned into 5‐μm sagittal sections by microtome and mounted onto glass slides for immunohistochemistry and imaging studies. Sections within approximately 300 μm of the right‐brain midline were used for immunohistochemistry. Nine age‐matched wild‐type mice were treated with vehicle and served as controls for the histopathology studies.

### 2.5 Immunohistochemistry

To detect degraded myelin, 5‐μm FFPE sagittal right brain sections were immunostained using a 1:1000 dilution of primary degraded myelin basic protein antibody (D‐MBP; rabbit‐anti‐mouse, Millipore Sigma Aldrich D‐MBP AB 5864) without antigen retrieval. This antibody preferentially detects debris of myelin degradation without cross‐reacting to normal myelin. Prior to antibody incubation, sections were neutralized for endogenous peroxidase and phosphatase with a 10‐minute incubation with Bloxall (Vector Laboratories SP‐6000; Newark, CA), rinsed twice with 0.1% Tween, Tris‐buffered saline, and blocked for non‐specific binding with 2.5% normal horse serum (Vector Laboratories S‐2012‐50). Blocking serum was replaced with primary antibody diluted in Antibody Diluent Reagent Solution (003218; Life Technologies), and sections were incubated at room temperature for 1 hour. Slides were washed and incubated 30 minutes with secondary alkaline phosphatase (AP)‐conjugated anti‐rabbit IgG (Vector ImPress‐AP; Vector Laboratories) with Vector Blue AP substrate as a chromagen. Slides were washed and counterstained with non‐specific Nuclear Fast Red to provide anatomical context. Slides were dehydrated, cleared, and coverslip‐mounted with Cytoseal 60 (Item 8310‐4; Thermo Fisher Scientific, South San Francisco, CA) prior to imaging.

To detect activated microglia, 5‐μm FFPE sagittal right brain sections were immunostained with 1:12000 dilution of primary microglial activation CD11b antibody (rabbit‐anti‐mouse; Abcam Item ab133357, Cambridge, MA) with heat‐induced antigen retrieval. CD11b antibody identifies inflammatory microglia that phagocytose damaged myelin and represent a secondary marker of myelin damage. Secondary antibody staining and detection were accomplished by the protocol described in the previous paragraph.

### 2.6 TUNEL assay for apoptosis

Click‐iT terminal deoxynucleotidyl transferase‐dUTP nick end labeling (TUNEL) was used to detect DNA fragmentation in situ according to the manufacturer's instructions (Item C10617; Thermo Fisher Scientific, South San Francisco, CA).^45^ Coverslips were mounted using Vectashield Vibrance Antifade hardening mounting medium (H‐1700; Vector Laboratories).

### 2.7 Microscopy

Imaging studies were conducted using an Echo Revolve fluorescence microscope (Echo, San Diego, CA). D‐MBP and CD11b immunofluorescence were visualized by the DAPI (UV) excitation/emission channel under 20× magnification. Non‐specific Nuclear Fast Red staining was visualized under the Texas Red excitation/emission channel for anatomical context in image overlays. TUNEL‐labeled apoptotic nuclei were visualized in the FITC excitation/emission channel under 4× magnification fields stitched together with Affinity Photo (Serif, Nottingham, UK).

### 2.8 Image quantification

Images were quantified manually using Image J software (v1.53q National Institutes of Health, Bethesda, MD) within the regions of interest for each mouse (unblinded to genotype and treatment; performed by a single investigator). D‐MBP staining in the parietal cortex and hippocampus was quantitatively scored. Quantification included both a 0.1 mm^2^ area of the CA3 region of the hippocampus, including the stratum pyramidale (SP), stratum oriens (SO), stratum lucidum (SL), and stratum radiatum (SR), and a 0.1 mm^2^ area of the adjacent parietal cortex above the corpus callosum for each mouse. For activated microglia expressing CD11b, a 20× field of the genu of the corpus callosum and the perforant pathway of the hippocampus was scored for each mouse. CD11b + microglia in the corpus callosum and hippocampus were scored semi‐quantitatively on a 0‐5 Likert scale (0, no activated microglia; 1, low numbers of activated microglia; 2, noticeably increased activated microglia; 3, intermediate activated microglia; 4, highly activated microglia; 5, very highly activated microglia). Scores in the genu of the corpus callosum and perforant pathway of the hippocampus were summed, for a maximum possible score of 10. Activated microglial cells were identified by unambiguous increase in microglial body size, ramification thickness, length (in rod‐shaped, activated microglial cells), clear phagosome extension, or amoeboid change in shape. TUNEL‐labeled apoptotic nuclei were quantified by manual counting of positive nuclei in Image J (v1.53q).

### 2.9 Survival study

*Scn1a* ^*+/−*^ mice were dosed by subcutaneous injection once daily between PND 7 through PND 35‐37 with fenfluramine (15 mg/kg; n = 17) or vehicle (n = 16). Wild‐type mice were injected with fenfluramine (n = 6) or vehicle (n = 9). Brain and plasma were harvested at PND 35‐37. An additional group of 24 *Scn1a* ^+/−^ mice was injected with 10 mg/kg/day diazepam; see Data S1 for further description.

#### 2.9.1 Survival monitoring

We maintained the *Scn1a* ^*+/−*^ mice and evaluated survival for an extended period of time in untreated animals (n = 99) before we injected treatment groups for the histological experiments. The untreated mice and all treatment groups were housed and maintained under identical conditions. We monitored and documented survival daily from PND 7 to PND 35‐37. Occasionally, we video‐recorded 3 *Scn1a* ^*+/−*^ mice using wide‐angle infrared cameras.

### 2.10 Statistical assessment

Survival was computed from PND 7 to PND 35‐37 or death. Quantitative differences in levels of the histopathological markers D‐MBP, CD11b +, and TUNEL among treatment groups were assessed using a 1‐way analysis of variance (ANOVA) with a post‐hoc Dunnett's test for multiple comparisons. Dunnett's tests were versus the vehicle‐treated DS mice control group. The survival statistics were evaluated using all *Scn1a* ^*+/−*^ mice, including untreated mice that were maintained in our laboratories earlier. Survival distributions of treatment groups were described using Kaplan–Meier methods, and the Logrank Mantel‐Cox test was used to assess differences in survival between treatment groups. *P*‐values <0.05 were considered statistically significant.

### 2.11 Diazepam treatment

In supplementary studies, 24 *Scn1a* ^*+/−*^ mice were treated with 10 mg/kg diazepam per Tiraboschi et al.^16^ (see Data S1). Survival was evaluated and compared with fenfluramine‐ or vehicle‐treated *Scn1a* ^*+/−*^ mice, untreated *Scn1a* ^*+/−*^ mice, and wild‐type controls. Immunohistochemistry with D‐MBP or CD11b antibodies and TUNEL assays for apoptosis were also conducted in surviving animals as described above and in the Data S1.

## 3 RESULTS

### 3.1 Demyelination

**FIGURE 1:**
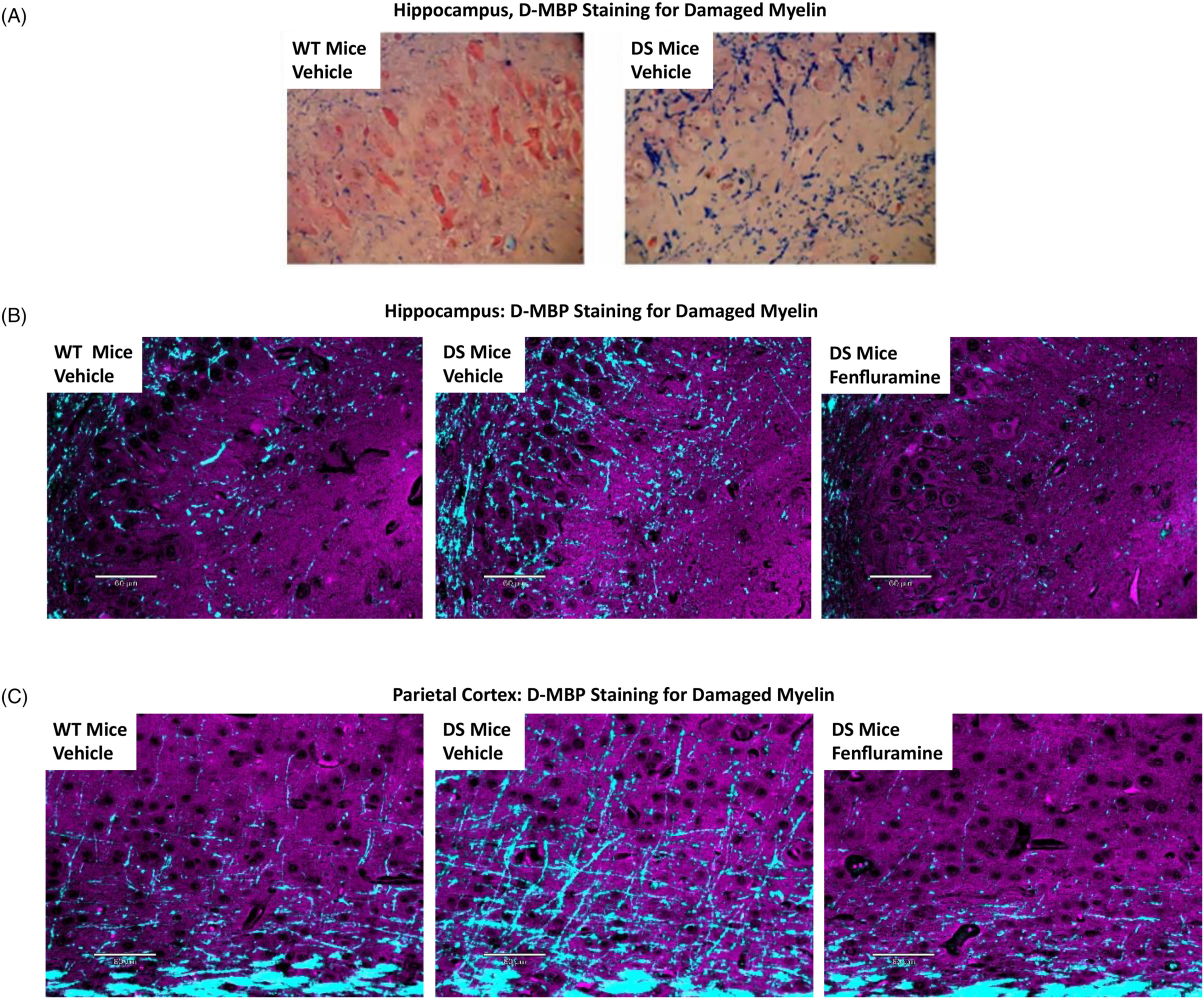
Damaged myelin by immunostaining in hippocampus (CA3) and parietal cortex in wild‐type mice (n = 9) or surviving *Scn1a* ^+/−^ Dravet syndrome mice (DS mice) treated subcutaneously with vehicle (n = 9) or 15 mg/kg/day fenfluramine (n = 13) from PND 7 to PND 35‐37. Representative images were taken of 5‐μm sagittal brain sections immunostained with D‐MBP. A, Hippocampus under visible light (40× magnification). B, Hippocampus under fluorescence. C, Parietal cortex under fluorescence. Under visible light, damaged myelin appears as spherical blue dots. In fluorescence images, cyan (DAPI channel, D‐MBP) indicates damaged myelin. Purple overlays (Texas Red channel, Nuclear Fast Red) provide anatomical context. Scale bars: 60 μm using a 20× objective. D‐MBP, degraded myelin basic protein; DS mice, *Scn1a* ^+/−^ Dravet syndrome mice; PND, postnatal day; WT, wild‐type.

FFPE sections for histopathology analysis were prepared for DS mice that completed treatment through PND 35‐37 in the survival study (vehicle, n = 9; fenfluramine, n = 13). Nine sections were prepared in parallel for wild‐type controls. Degenerated myelin was assessed in brain sections from DS mice with D‐MBP staining and scoring under visible light and fluorescence microscopy (Figure 1). Representative sections demonstrated evidence for increased myelin damage in the hippocampus, as indicated by a qualitative increase in spherical blue dots in DS mice relative to wild‐type controls (Figure 1A). In general, these sections showed less visibly intact linear myelin cross‐detected by the D‐MBP antibody than wild‐type controls.

Overall, microscopic evaluation of the sagittal sections revealed that the regions with the most D‐MBP immunofluorescence were the hippocampus and parietal cortex (representative images, Figure 1B,C, respectively). In these regions, spheroidal and punctate debris was detected by the D‐MBP antibody. For quantitative image analysis, these regions were combined.

**FIGURE 2:**
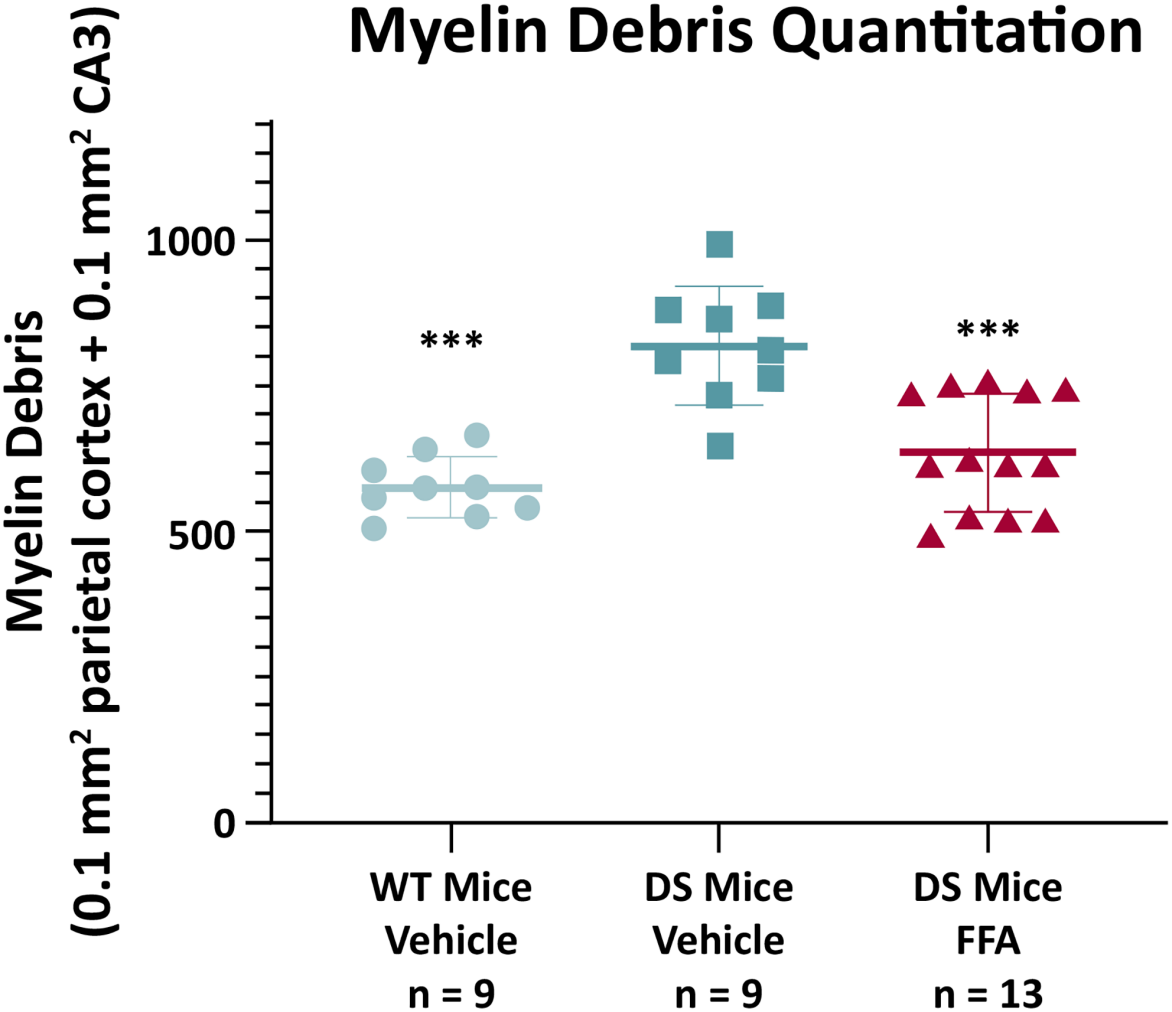
Damaged myelin quantitation in parietal cortex and hippocampus CA3. Myelin debris was quantified by fluorescence microscopy in 5‐μm sagittal brain sections via image quantification with D‐MBP antibody immunostaining. Data are plotted as mean ± SD. *P*‐values were calculated by ANOVA with post‐hoc Dunnett's test for multiple comparison. ANOVA results: *P* < 0.0001. Dunnett's multiple comparisons test results compared with DS mice, vehicle‐treated group: ***P < 0.001 (left to right, vs DS Mice Vehicle: WT Mice Vehicle, *P* < 0.0001; DS mice FFA, *P* = 0.0002). ANOVA, analysis of variance; DS mice, *Scn1a* ^*+/−*^ mouse model of Dravet syndrome; FFA, fenfluramine; WT, wild‐type.

Image quantification of myelin debris per unit area (0.1 mm^2^ of the CA3 region of the hippocampus and 0.1 mm^2^ parietal cortex) demonstrated that the level of degenerated myelin was significantly higher in the DS mice (818 ± 101; ANOVA, *P* < 0.0001) compared to wild‐type mice (575 ± 53, *P* < 0.0001 by Dunnett's post‐hoc test for multiple comparisons; Figure 2). Treatment with fenfluramine significantly reduced the level of damaged myelin in DS mice (635 ± 101) compared to vehicle‐treated DS mice (*P* = 0.0002 by Dunnett's test; Figure 2).

### 3.2 Activated microglia

**FIGURE 3:**
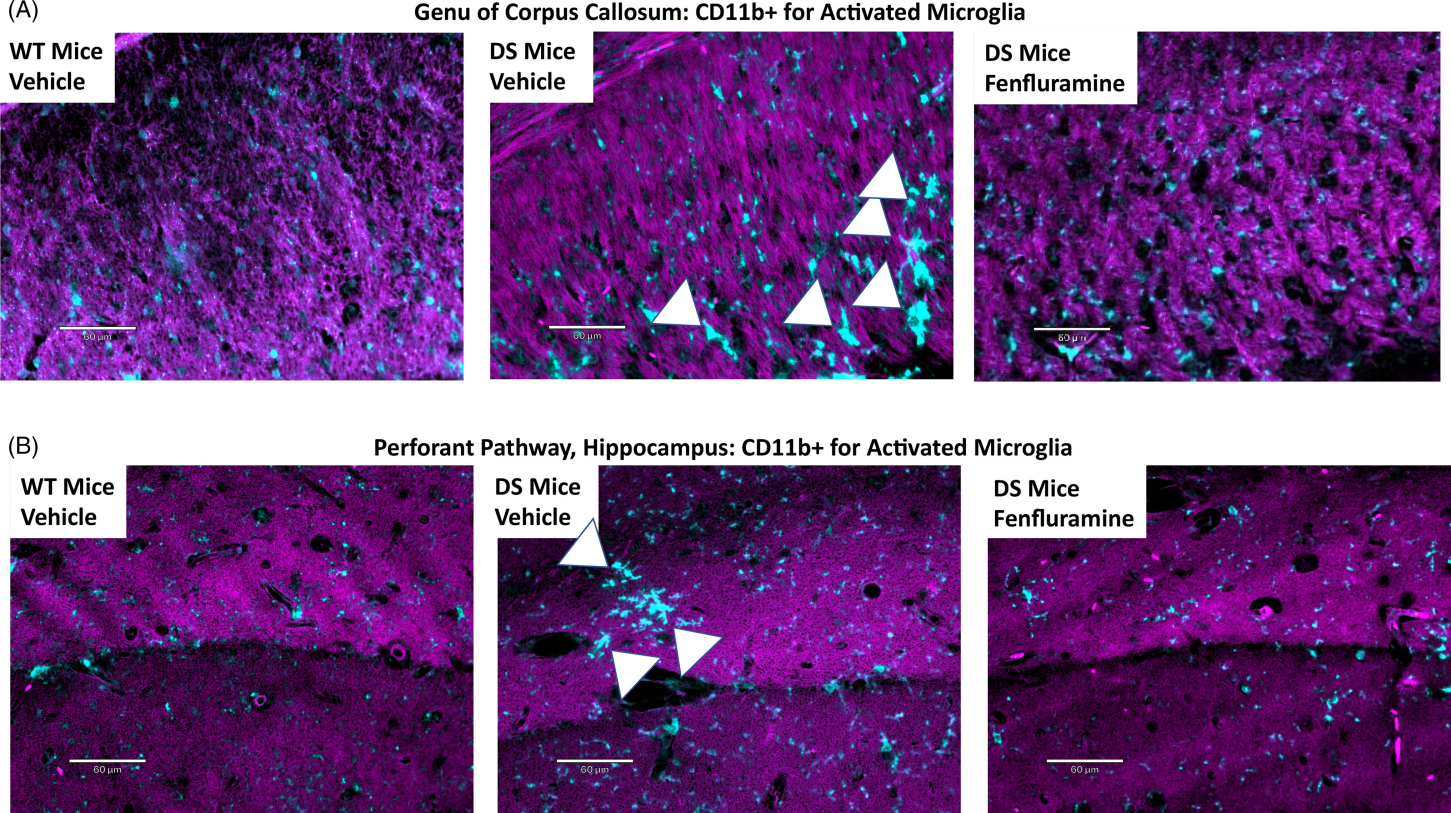
Activated microglia by CD11b + immunostaining in corpus callosum (genu) and hippocampus (perforant pathway) in wild‐type mice (n = 9) or surviving DS mice treated subcutaneously with vehicle (n = 9) or 15 mg/kg/day fenfluramine (n = 13) from PND 7 to PND 35‐37. Representative images were taken of 5‐μm sagittal brain sections immunostained with CD11b + antibody for activated microglia. A, Genu of the corpus callosum. B, Perforant pathway of the hippocampus. Cyan (DAPI channel, CD11b) represents CD11b + activated microglia; arrowheads indicate canonical activated microglia (note elongated, rod shape with clear phagosome extension). Purple overlays (Fast Red staining, Texas Red channel) provide neuroanatomical context. Scale bars: 60 μm using a 20× objective. DS, Dravet syndrome; PND, postnatal day; WT, wild‐type.

Activated CD11b + microglia were more apparent in the genu of the corpus callosum and the perforant pathway of the hippocampus (Figure 3A,B, respectively) than any other brain region. Sections from the vehicle‐treated DS mice were enriched in clearly elongated, rod‐shaped microglia with clear phagosome extension (central panel; note arrowheads) compared to wild‐type mice (left panels).

**FIGURE 4:**
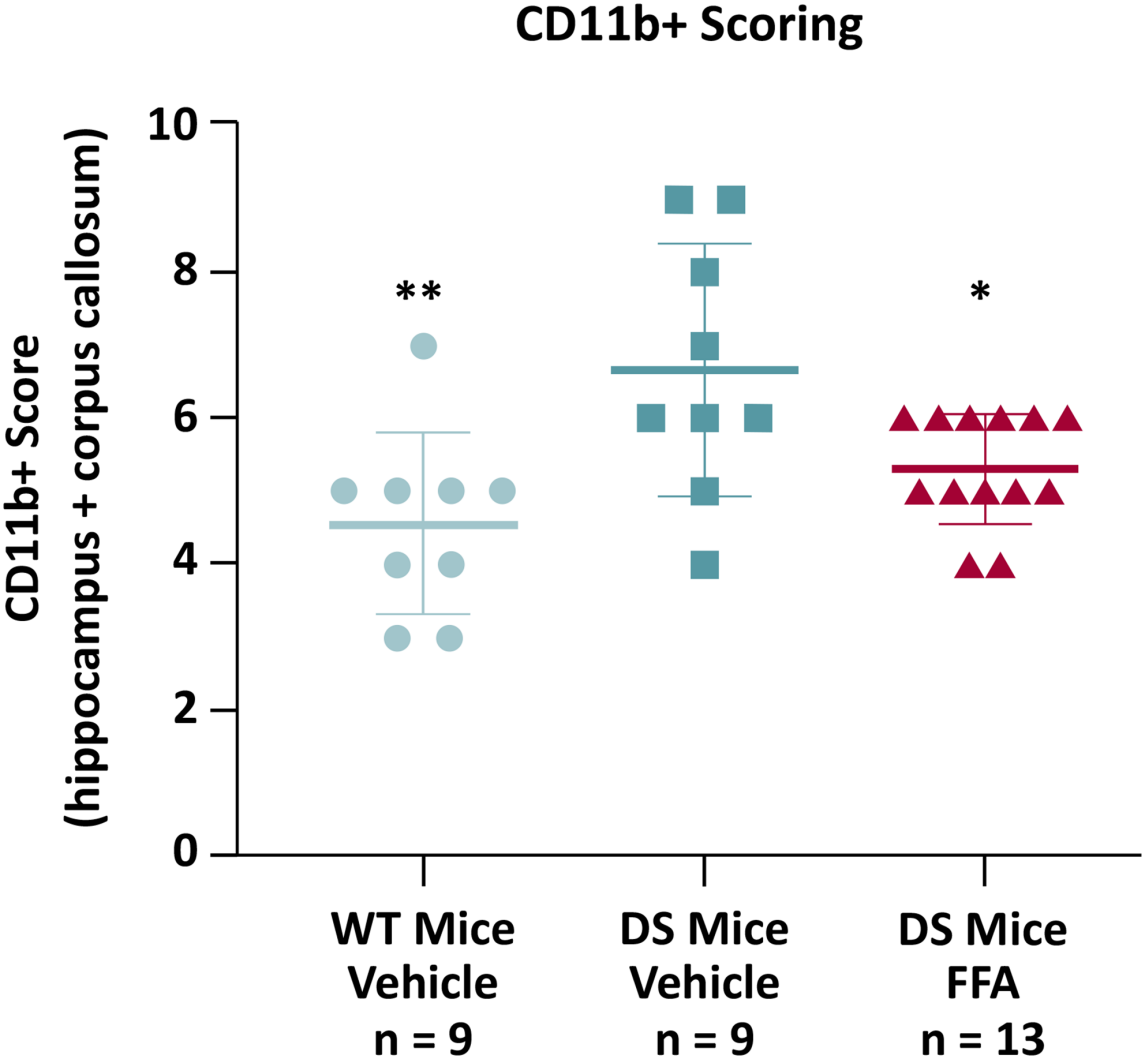
Image quantification of the effect of fenfluramine treatment on CD11b + immunostaining of microglia in the hippocampus and corpus callosum. Images (one 5‐μm sagittal section per mouse) were evaluated semi‐quantitatively on a Likert scale of 0‐5 (0, no activated microglia; 5, highly activated microglia). Data are plotted as mean ± SD; *P*‐values were calculated by ANOVA with post‐hoc Dunnett's test for multiple comparison. ANOVA results: *P* = 0.0040. Dunnett's multiple comparisons test results compared with DS mice, vehicle‐treated group: **P < 0.01; *P < 0.05 (left to right, vs DS Mice Vehicle: WT Mice Vehicle, *P* = 0.0022; DS mice FFA, *P* = 0.0317). ANOVA, analysis of variance; DS mice, *Scn1a* ^+/−^ Dravet syndrome mice; FFA, fenfluramine; WT, wild‐type.

Average (±SD) pathology scores showed that activated CD11b + microglia were enriched in vehicle‐treated DS mouse corpus callosum and hippocampus compared with wild‐type mice (ANOVA: *P* = 0.0040; 6.67 ± 1.73 vs 4.56 ± 1.24; *P* = 0.0022 by Dunnett's test; Figure 4). Treatment with fenfluramine significantly reduced the levels of CD11b + microglia in DS mice compared to vehicle‐treated DS mice (5.31 ± 0.75 vs 6.67 ± 1.73; *P* = 0.0317 by Dunnett's test; Figure 4).

### 3.3 Apoptosis

**FIGURE 5:**
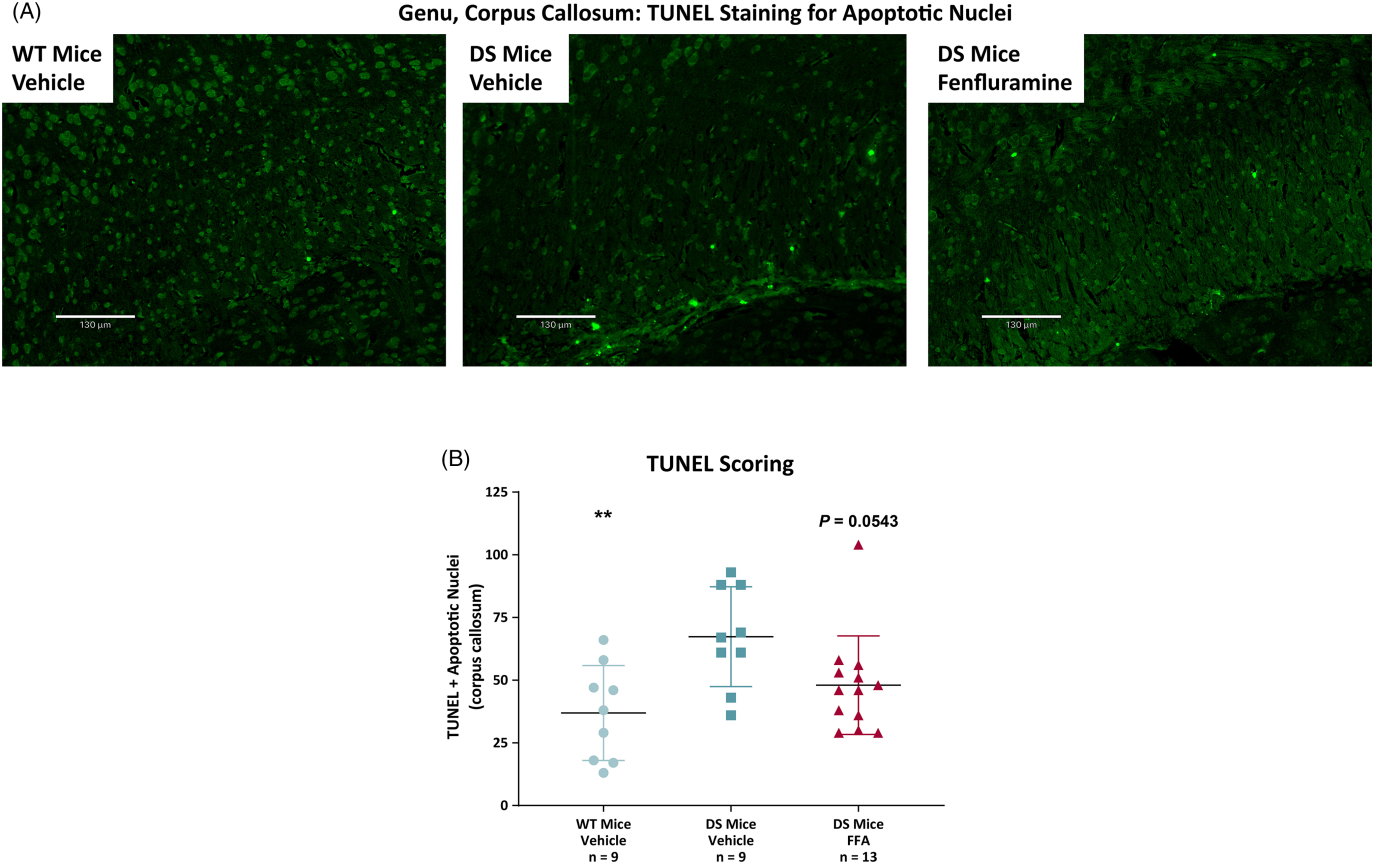
Apoptosis by TUNEL staining in 5‐μm sagittal sections of the corpus callosum in wild‐type mice or surviving DS mice treated subcutaneously once daily with vehicle (n = 9) or 15 mg/kg fenfluramine (n = 13) from PND 7 to PND 35‐37. A, Representative images from genu of the corpus callosum (scale bar, 130 μm; green [FITC] channel, 10× objective) and (B) manual image quantification of apoptotic nuclei by TUNEL using 4× magnification fields stitched together with Affinity Photo. In B, data are plotted as mean ± SD; *P*‐values were calculated by ANOVA with post‐hoc Dunnett's test for multiple comparison. ANOVA results: *P* = 0.0087. Dunnett's multiple comparisons test results compared with DS vehicle‐treated group: **P < 0.01 (left to right, vs DS Mice Vehicle: WT Mice Vehicle, *P* = 0.0049; DS mice FFA, *P* = 0.0543). ANOVA, analysis of variance; DS mice, *Scn1a* ^+/−^ Dravet syndrome mice; FFA, fenfluramine; PND, post‐natal day; TUNEL, terminal deoxynucleotidyl transferase biotin‐dUTP nick end labeling; WT, wild‐type.

Epileptogenesis is associated with increased neuronal apoptosis,^26^ including in preclinical models of DS.^46^ Sagittal section specimens from all treatment groups were evaluated for apoptosis by TUNEL assays to detect apoptotic DNA fragmentation. Hippocampus, corpus callosum, cortex, and all three combined were evaluated quantitatively. Intensely fluorescent apoptotic nuclei were most abundant in the corpus callosum (47.1 ± 22.4) relative to hippocampus (4.7 ± 3.0) and cortex (9.3 ± 7.3). Quantification of apoptotic nuclei indicated that DS mice had significantly more TUNEL fluorescence (67.3 ± 19.9; ANOVA: *P* = 0.0087) than vehicle‐treated wild‐type controls (36.9 ± 18.9; *P* = 0.0049 by Dunnett's test) (Figure 5). Pretreatment of DS mice with 15 mg/kg/day fenfluramine resulted in a reduction of TUNEL fluorescence in the corpus callosum (48.0 ± 19.6) relative to vehicle‐treated DS mice (67.3 ± 19.9; *P* = 0.0543 by Dunnett's test).

### 3.4 Survival

**FIGURE 6:**
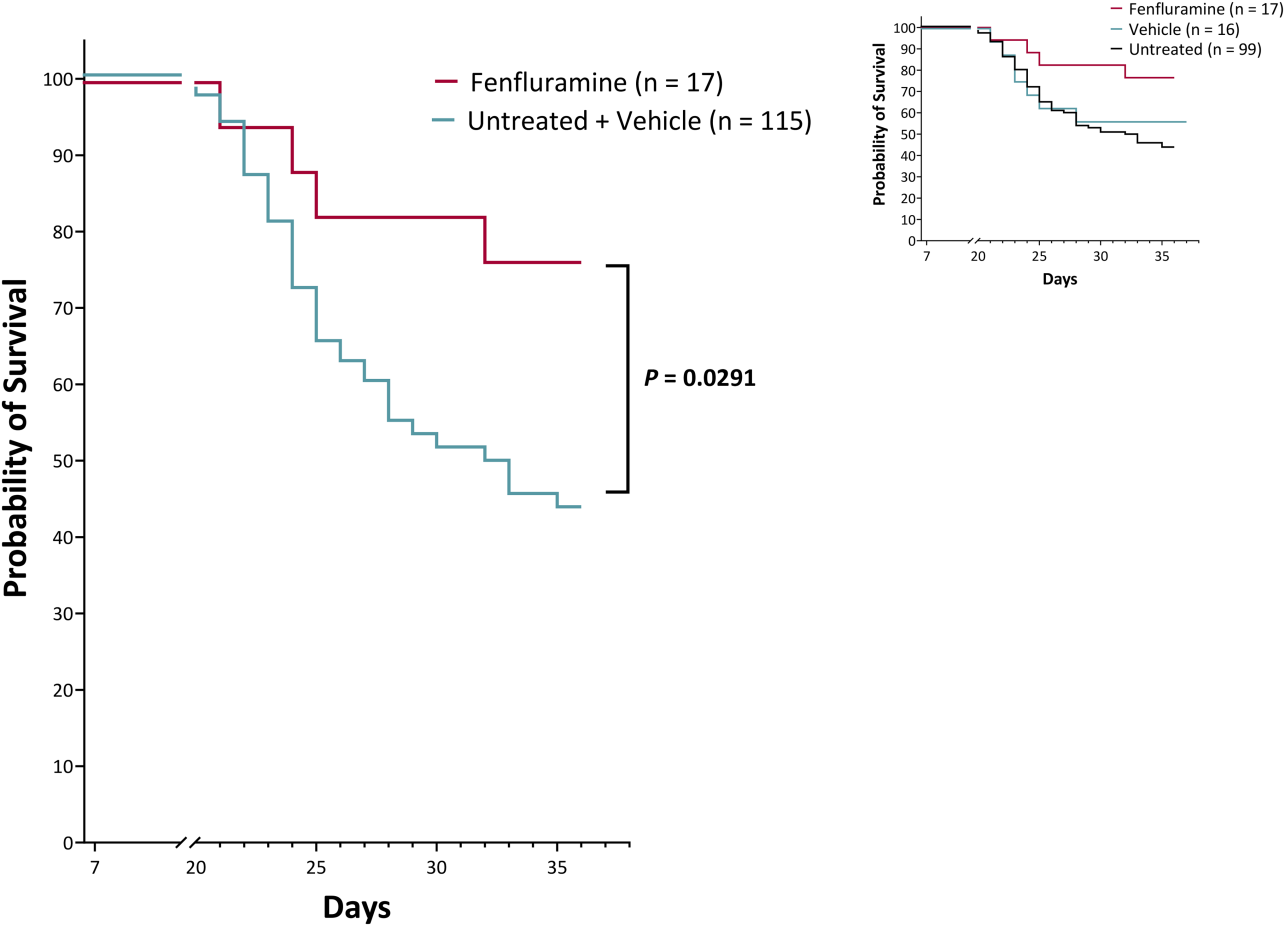
Effect of treatment on survival of *Scn1a* ^+/−^ Dravet syndrome mice (DS mice). DS mice treated subcutaneously once daily with vehicle or no treatment (n = 115) or 15 mg/kg fenfluramine (n = 17) from postnatal day (PND) 7 to 35‐37. Survival was described using Kaplan–Meier methods. The Logrank Mantel‐Cox test was *P* = 0.0291 for the combined vehicle + untreated group versus fenfluramine. Inset compares untreated (n = 99), vehicle (n = 16), and fenfluramine (n = 17) groups (*P* = 0.0719).

The dosing period (PND 7 to PND 35‐37) represented an aggregate of 28‐30 days of once‐daily dosing per treatment group. Across all treatment groups during the entire treatment period, an increase in mortality was observed in DS mice, beginning at approximately PND 21 (Figure 6). By PND 35‐37, 55% (63/115) of the untreated or vehicle‐treated *Scn1a* ^*+/−*^ mice had died (Figure 6; 44% [7/16] of the vehicle‐treated mice had died [see Figure 6, inset]), consistent with published reports describing approximately 50% lethality by age 1 month in the B6x129(F1.*Scn1a* ^*+/−*^) Dravet mouse model.^24^ In the fenfluramine treatment group of DS mice, mortality was 24% (4/17), representing a significant reduction in mortality relative to untreated or vehicle‐treated control DS mice (*P* = 0.0291, Logrank Mantel‐Cox test; Figure 6). In parallel with the DS mice, wild‐type control groups were injected with vehicle (n = 9) or fenfluramine (n = 6); no deaths were observed.

DS mice exhibited spontaneous seizures. Video recordings were available from three DS mouse controls (Data S1, Videos [Link], [Link]); all three mice died immediately following a convulsive seizure rated 5 on the Racine seizure rating scale.^47^ Body position at time of death showed hindlimb extension at 180 degrees (see reference^48^) in all three recordings.

## 4 DISCUSSION

This study is the first to demonstrate that chronic treatment with fenfluramine in DS mice (a) reduces levels of myelin degeneration, microglia activation/neuroinflammation, and apoptosis and (b) increases survival. The effects of fenfluramine were most pronounced in the parietal cortex and CA3 of the hippocampus for demyelination, in the corpus callosum (genu) and perforant pathway of the hippocampus for activated microglia, and in the corpus callosum for apoptosis.

### 4.1 Fenfluramine impact on myelin degradation

Our study provides the first evidence in a DS mouse model that fenfluramine reduces neuronal markers of myelin damage. Atypical myelinogenesis in the corpus callosum was evident before seizure onset in another *Scn1a* mouse model of DS.^25^ White matter (myelin) damage is a common pathology in animal models of epilepsy and in patients with epilepsy and persons with demyelinating motor diseases (e.g., multiple sclerosis).^30^, ^31^, ^49^ Further, the spheroidal D‐MBP staining we observed in the DS mouse hippocampus is consistent with clinical studies in patients with DS showing elevated white matter damage throughout the brain by MRI^27^, ^50^ and upon autopsy.^29^

It is unclear whether myelination protection/repair is a secondary consequence of reduced seizure activity by fenfluramine, whether fenfluramine acts through a direct mechanism that either protects or repairs myelin, or a combination of both. Myelin damage is a general consequence of epilepsy.^28^, ^29^ However, fenfluramine's dual‐action, serotonergic and sigma‐1 receptor mechanism of action is unique among antiseizure medications.^51^ A growing body of evidence supports a combination of serotonergic and Sigma1R positive modulatory activity in pro‐survival, pro‐cognitive effects of fenfluramine in addition to seizure control.^51^ Some evidence suggests that this unique pharmacology may also play a direct role in reducing neuroinflammation and/or protecting or repairing myelin.^52^, ^53^, ^54^ Sigma1R was identified as a critical regulator of inflammation in a knockout Sigma1R mouse model,^52^ and serotonin reduced levels of inflammatory cytokines in brain homogenates of rats after pentylenetetrazole‐induced seizures.^55^ Sigma1R regulates myelination in the brain, with defects associated with neurodegenerative disorders (e.g., multiple sclerosis).^53^, ^54^, ^56^ In a mouse model of spatial learning and memory, fenfluramine positively modulated Sigma1R to prevent dizocilpine‐induced amnesia,^15^ and later reports suggested that the neuro (active) steroids dehydroepiandrosterone sulfate and pregnenolone sulfate were endogenous ligands mediating these neuroprotective effects.^14^ Activation of Sigma1R by the neurosteroid dehydroepiandrosterone contributed to hippocampal neurogenesis in neurodegenerative mouse models.^56^ Whether the dual‐action serotonergic/Sigma1R pharmacology of fenfluramine contributes to preventing myelin damage in DS mice remains to be established.

### 4.2 Activated microglia

To our knowledge, this is the first report of fenfluramine decreasing activated CD11b + microglia in a mammalian DS model. CD11b + microglia engulf and recycle cellular debris, including degraded myelin. Reduction of CD11b + immunofluorescence after fenfluramine may reflect preserved myelin integrity. Moreover, CD11b immunostaining was specifically selected because it is a marker of active microglia with a phagocytic phenotype.^57^ CD11b detects neuroinflammation. A reduction in CD11b + microglia suggests reduced neuroinflammation and myelin damage after fenfluramine treatment. It is unclear whether these effects are a result of reduced seizure activity or a direct result of fenfluramine on neuroinflammation in these brain regions. The differences observed in the neuroanatomical location of the fenfluramine effect on demyelination (CA3 of hippocampus and parietal cortex) and microglia (perforant pathway of hippocampus and genu of corpus callosum) also remain to be investigated.

Neuroinflammation in the context of oxidative stress has been described in immunohistochemistry studies of resected brain tissue of patients with epileptic cortical malformations, including tuberous sclerosis complex (cortical tubers), hemimegalencephaly, and focal cortical dysplasia.^58^ Further, white matter myelin damage was reported by myelin immunohistochemistry in resected brain tissue from patients with tuberous sclerosis complex or focal cortical dysplasia.^59^ The functional consequences of reduced neuroinflammation after fenfluramine remain to be investigated.

### 4.3 Apoptosis

The observation that fenfluramine inhibited apoptosis in DS mice is a novel finding, but the implications remain to be established. There is some evidence that apoptosis impacts the excitatory/inhibitory imbalance in both seizures and non‐seizure comorbidities.^26^, ^46^ A reduction in pathological apoptosis long‐term may afford better seizure control and improved cognition, as demonstrated after administration of liraglutide in an *Scn1a* knockout model.^26^ Apoptotic neurodegeneration has also been cited as a mechanism for worsening cognition after treatment of various antiseizure medications.^60^ Patients with DS are typically taking multiple concomitant antiseizure medications.^61^ Some antiseizure medications inhibit endogenous neuroprotection mechanisms in the brain, resulting in reduced brain mass and cognitive impairment. Fenfluramine, by contrast, improved the regulation of behavior, cognition, and emotions in clinical studies in patients with DS.^13^, ^62^ The amelioration of apoptosis in the corpus callosum in our study by fenfluramine could be a result of restoring neuroprotective mechanisms, such as neurotrophin systems critical for neuronal survival.^60^ Alternatively, apoptosis may play a role in structural remodeling of the CNS architecture in specific brain regions. Prior reports have demonstrated the importance of remodeling of neurons, astrocytes, and glia in impacting neuronal survival, pruning, and dendritic outgrowth.^63^ This hypothesis has some support in a zebrafish *scn1lab* ^*mut/mut*^ DS model where fenfluramine preserved the architectural integrity of inhibitory GABAergic neurons.^16^ Further experiments are needed to determine whether the impact of fenfluramine on apoptosis in the corpus callosum plays a role in neuroprotection, neuronal remodeling, anti‐inflammation, and/or a combination of these and other mechanisms.

### 4.4 Survival

Our study provides the first evidence that fenfluramine promotes survival in a DS mouse model. The enhanced survival with fenfluramine in DS mice is consistent with a report of lower all‐cause mortality and SUDEP rates (both rates were 1.7 per 1000 person‐years in 732 patients with DS who were treated with fenfluramine over a total of 1185.3 person‐years of exposure) compared with a published cohort study in patients who were not receiving fenfluramine^64^ (all‐cause mortality rates of 15.8 deaths per 1000 person‐years and SUDEP rates of 9.3 per 1000 person‐years over a total of 1073 person‐years).^64^ Further experimentation is required to determine whether the improved survival after fenfluramine we observed in our study is linked to reduction in SUDEP. Evidence from an audiogenic seizure DBA/1 mouse model showed that fenfluramine treatment reduced risk of convulsive seizure‐induced respiratory arrest (S‐IRA) preceding SUDEP at concentrations which had no impact on total seizure threshold (15 mg/kg fenfluramine for 8 h).^17^ The effect of fenfluramine was more potent at blocking S‐IRA than seizures at this dose, and was inhibited by serotonergic 5‐HT_4_ receptor antagonists.^21^ The authors concluded that seizures and S‐IRA likely followed related but non‐overlapping mechanisms, and that fenfluramine acted pharmacologically on both pathways. In prior studies with DS mice, SUDEP occurs immediately following convulsive seizures, and conditional knockout of *Scn1a* in brain recapitulated the SUDEP phenotype.^36^ Although the three videos of the animals that died in our study are consistent with a convulsive seizure prior to sudden death, video recording of all animals is needed for definitive proof.

### 4.5 Limitations

This study has some limitations. First, seizure activity was not quantified in this study, nor was continuous, 24‐hour video surveillance conducted on all animals. Our study reports all‐cause mortality; additional studies would be needed using electroencephalogram (EEG) and continuous video surveillance on all study animals to determine whether all deaths were preceded by a convulsive seizure, as well as the impact of fenfluramine on the number, intensity, and duration of seizures. Second, the fenfluramine dose chosen (15 mg/kg/day) prevented S‐IRA preceding SUDEP in the DBA/1 mouse model without significantly affecting the seizure threshold.^17^ Additional experiments are needed to determine whether fenfluramine also inhibits S‐IRA in the DS *Scn1a* ^*+/−*^ mouse model, and to determine the correlation between the pro‐survival effect of 15 mg/kg/day fenfluramine and both seizure threshold and neuro‐histopathology. Third, the neuroimaging results reported in this study are limited by survivor bias. Additional studies are needed to evaluate neural histopathology immediately after death to determine whether histopathology differed in the mice that died compared to survivors. Finally, our study reports the use of fenfluramine as monotherapy, whereas clinical settings use fenfluramine as an add‐on medication to existing regimens; further studies are needed to determine the effect of fenfluramine in combination with routinely used antiseizure medications on survival and markers of neuroinflammation in mouse models of DS.

### 4.6 Future directions

Further studies are warranted to confirm and extend our neuroinflammation results. Electron microscopy is needed to quantify G‐ratio to better assess whether demyelination is ongoing in Dravet mice, and to assess the effects of fenfluramine on this endpoint. High‐magnification, high‐resolution confocal microscopy images can better assess morphological changes in CD11b + activated microglial cells. Additional apoptosis markers are needed to corroborate the TUNEL staining (e.g., Bax, Bcl2, cleaved caspase), with high‐resolution confocal imaging to identify apoptotic cell types using double immunofluorescence (e.g., main neuronal/glial cell types and apoptotic markers).

Our survival results warrant future studies to investigate the mechanisms behind the observed pro‐survival outcome of fenfluramine treatment. Most importantly, obtaining seizure counts (number and intensity) and EEG recordings is an important next step in understanding the mechanism behind how 15 mg/kg/day fenfluramine improves survival in *Scn1a* ^*+/−*^ mice. Recently published studies investigating outcomes of novel antiseizure medications in the *Scn1a* ^*+/−*^ mouse model provide the experimental framework for future studies (see Hawkins et al.^65^ for a recent example), including behavior testing, pharmacokinetics to confirm brain and plasma exposure and compare subcutaneous versus intraperitoneal routes of administration, continuous video surveillance with EEG recordings, and Racine scoring for seizures.^17^

## 5 CONCLUSIONS

Our study is the first to report that fenfluramine treatment of DS mice reduces neuroinflammation and demyelination—most notably in the hippocampus, corpus callosum, and parietal cortex—and increases survival. This study provides potential neuropathological pathways for the impact of fenfluramine on promoting survival and reducing neuroinflammation, and is hypothesis‐generating for insights into the neuroanatomical structure–function relationships. Further studies are needed to determine whether the histopathological changes following fenfluramine are related to decrease in SUDEP‐related mortality and whether pro‐survival is solely secondary to seizure control, or whether additional mechanisms are involved.

## AUTHOR CONTRIBUTIONS

Dr. Cha, Dr. Filatov, Dr. Smith, and Dr. Reeder participated in (a) experimental design, data acquisition, and analysis/interpretation of the data and (b) drafting and critically revising the article with respect to intellectual content. Dr. Gammaitoni participated in (a) experimental design and analysis/interpretation of the data and (b) drafting and critically revising the article with respect to intellectual content. Dr. Lothe participated in (a) interpretation of the data and (b) critically revising the article with respect to intellectual content. All authors gave final approval of the manuscript.

## CONFLICT OF INTEREST STATEMENT

Dr. Cha did not have conflicts of interest outside of employment at Zogenix, Inc, now a part of UCB, at the time this study was conducted. Dr. Filatov, Dr. Smith, Dr. Gammaitoni, and Dr. Reeder were employees of and/or had ownership interest in Zogenix at the time this study was conducted. Dr. Lothe is an employee of UCB Pharma and discloses ownership interest.

## ETHICAL STATEMENT

We confirm that we have read the Journal's position on issues involved in ethical publication and affirm that this report is consistent with those guidelines.

## Supporting information


Data S1.

Video S1.

Video S2.

Video S3.

## Data Availability

Data from non‐clinical studies are outside of UCB's data sharing policy and are unavailable for sharing.
