# Supplementary material for: Fenfluramine increases survival and reduces markers of neurodegeneration in a mouse model of Dravet syndrome
Source: Epilepsia Open. 2023 Dec 22;9(1):300–13. doi: 10.1002/epi4.12873 (PMC10839300; doi:10.1002/epi4.12873)
Supplement: Supplementary file 1 — Data S1. [file EPI4-9-300-s004.docx]

**SUPPORTING INFORMATION**

In a homozygous mutant *scn1lab^mut/mut^* zebrafish model of Dravet syndrome (DS), Tiraboschi et al. (2020) compared immunohistochemistry and seizure endpoints in zebrafish exposed to vehicle, 50 µM fenfluramine, or 10 µM diazepam from 3 days postfertilization (dpf) to 6 dpf. Diazepam, an antiseizure medication (ASM) commonly used clinically as a rescue medication, served as an ASM control. Diazepam was chosen as a comparator to match previous study designs in this *scn1lab* mutant zebrafish model of DS, which is orthologous to the human *SCN1A*,^1^ and because of clinical use as a rescue medication (eg, in status epilepticus).^2-4^ We reproduced this experimental design in our *Scn1a^+/-^* mouse model in supporting experiments, using 10 mg/kg diazepam based on the zebrafish dosing paradigm. In these experiments, we cannot rule out that long-term, high-dose diazepam may result in tolerance and reduced efficacy over time in our experimental paradigm. For completeness, we present the results of the diazepam group data as supporting data.

***Demyelination***

The demyelination experiments were performed as described in sections 2.5 and 3.1, including a diazepam group (10 mg/kg; n = 9). In the hippocampus CA3 region and parietal cortex, treatment with diazepam had no effect on D-MBP levels (751 ± 157) relative to DS vehicle-treated mice (*P* > 0.05 [*P* = 0.4136] by Dunnett’s test for multiple comparisons; **Figure S1 and S2**).

***Activated microglia***

The activated microglia experiments were performed as described in sections 2.5 and 3.3, including a diazepam group (10 mg/kg; n = 9). Sections from the genu of the corpus callosum and perforant pathway of the hippocampus in diazepam-treated DS mice showed evidence of microglial activation that was comparable to sections from vehicle-treated DS mice (compare lower right and upper right panels of **Figures S3A and S3B**) Diazepam treatment had no quantifiable effect on levels of CD11b+ microglia compared to vehicle treatment in DS mice (6.67 ± 0.87 vs 6.67 ± 1.73; *P* > 0.05 [*P* > 0.9999 by Dunnett’s test; **Figure S4**]).

***Apoptosis***

The apoptosis experiments (TUNEL staining) were performed as described in sections 2.6 and 3.3, including a diazepam group (10 mg/kg; n = 9). Pretreatment of DS mice with either fenfluramine 15 mg/kg/day or diazepam 10 mg/kg/day resulted in a reduction in TUNEL fluorescence (fenfluramine: 48.0 ± 19.6; diazepam: 35.9 ± 20.0) relative to vehicle-treated DS mice (67.3 ± 19.9; *P* = 0.0733 vs fenfluramine; *P* = 0.0045 vs diazepam by Dunnett’s test) (**Figure S5**).

***Survival***

The survival experiments were performed as described in sections 2.9 and 3.4, including a diazepam group (10 mg/kg; n = 24). In the diazepam treatment group of DS mice, 62% (15/24) of the DS mice had died by PND 35-37; this proportion was numerically higher than combined untreated and vehicle-treated control DS mice (63/115; 55% [untreated and vehicle combined]) and the fenfluramine-treated group (4/17; 24%), but the difference did not meet the significance threshold by Logrank Mantel-Cox test (*P* = 0.0579; *P* = 0.1035 when the untreated [56/99, 57%] and vehicle [7/16; 44%] groups were treated independently; see **Figure S6, inset**).

**SUPPORTING INFORMATION—FIGURES**

**Figure S1.** Damaged myelin by immunostaining in hippocampus (CA3) and parietal cortex in wild-type mice (n = 9) or surviving DS mice treated subcutaneously with vehicle (n = 9), 15 mg/kg/day fenfluramine (n = 13), or 10 mg/kg/day diazepam (n = 9) from PND 7 to PND 35-37. Representative images were taken of 5-µm sagittal brain sections immunostained with D-MBP. (A) Hippocampus under fluorescence. (B) Parietal cortex under fluorescence. Under visible light, damaged myelin appears as spherical blue dots. In fluorescence images, cyan (DAPI channel, D-MBP) indicates damaged myelin. Purple overlays (Texas Red channel, Nuclear Fast Red) provide anatomical context. Scale bars: 60 µm using a 20X objective. D-MBP, degraded myelin basic protein; DS mice, *Scn1a*^+/-^ Dravet syndrome mice; PND, postnatal day; WT, wild-type.

**
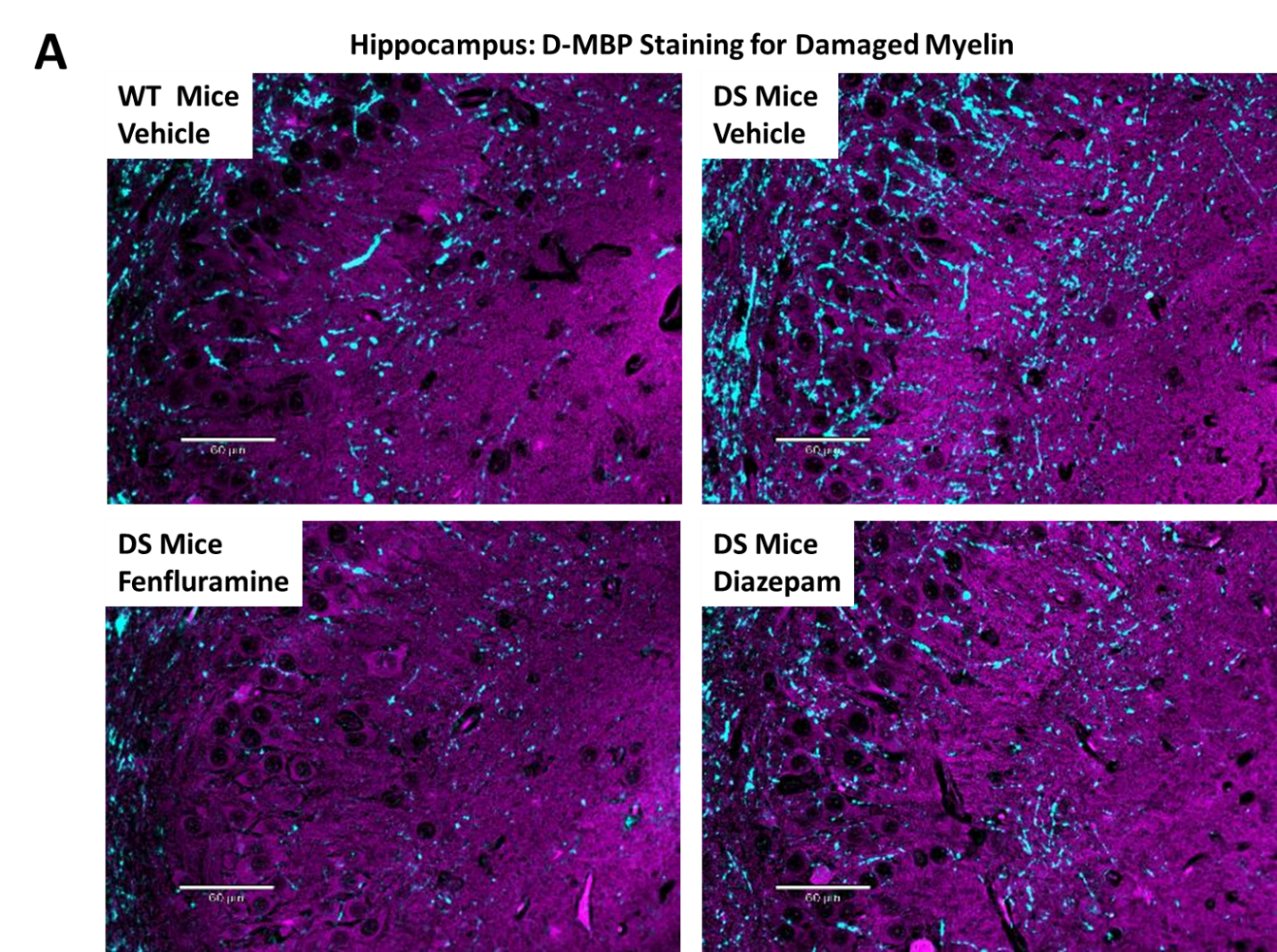
**

**
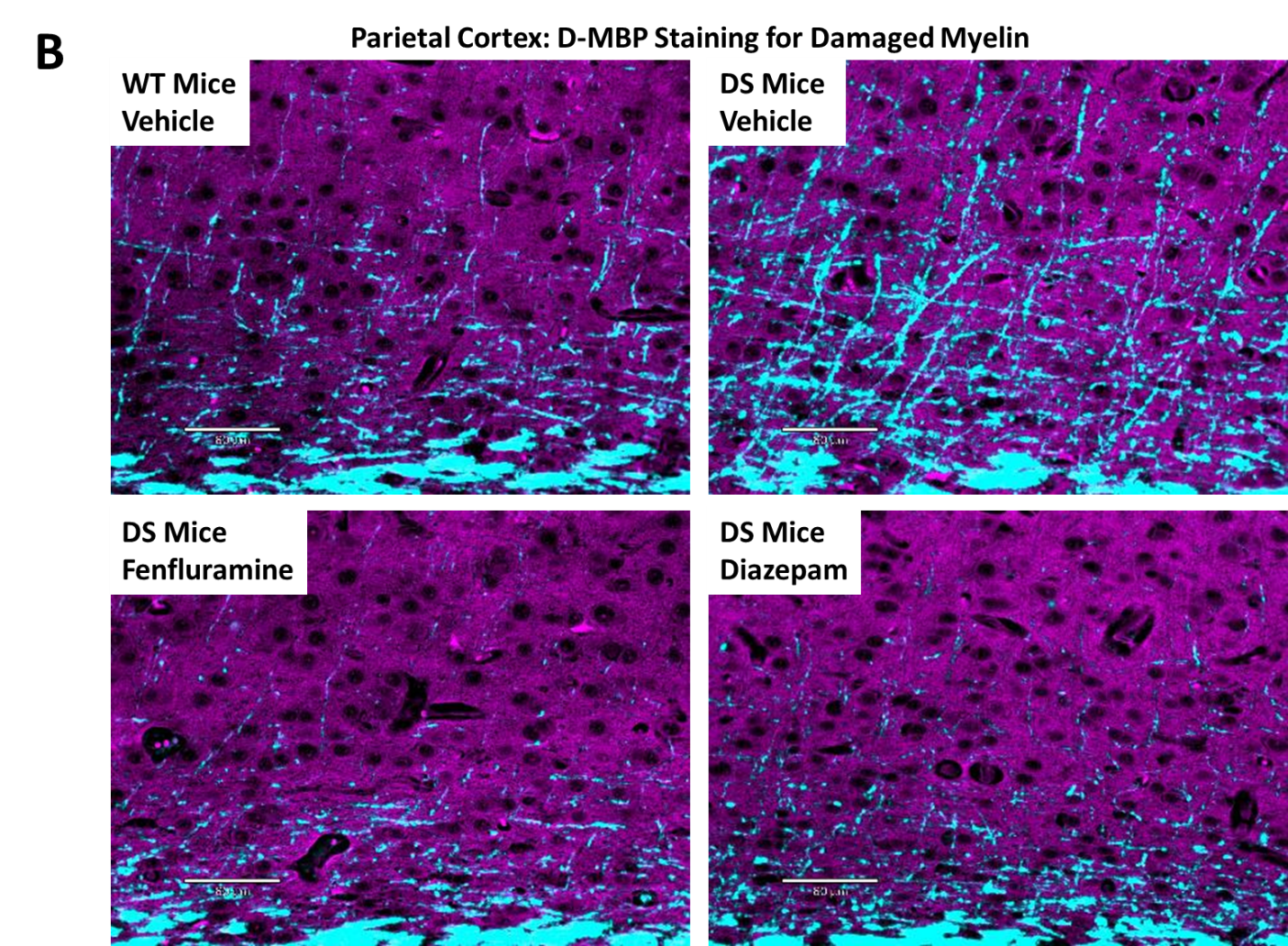
**

**Figure S2.** Damaged myelin quantitation in parietal cortex and hippocampus CA3. Myelin debris was quantified by fluorescence microscopy in 5-µm sagittal brain sections via image quantification with D-MBP antibody immunostaining. Data are plotted as mean ± SD; *P*-values were calculated by ANOVA with post-hoc Dunnett’s test for multiple comparison. ANOVA results: *P* = 0.0002; Dunnett’s multiple comparisons test results compared with DS vehicle-treated group: *** *P* < 0.001; ** *P* < 0.01; ns *P* > 0.05 (left to right, vs DS Mice Vehicle: WT Mice Vehicle, *P* = 0.0003; DS mice FFA, *P* = 0.0012; DS Mice Diazepam, *P* = 0.4136). ANOVA, analysis of variance, DS mice, *Scn1a^+/-^* mouse model of Dravet syndrome; FFA, fenfluramine; WT, wild-type.


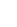

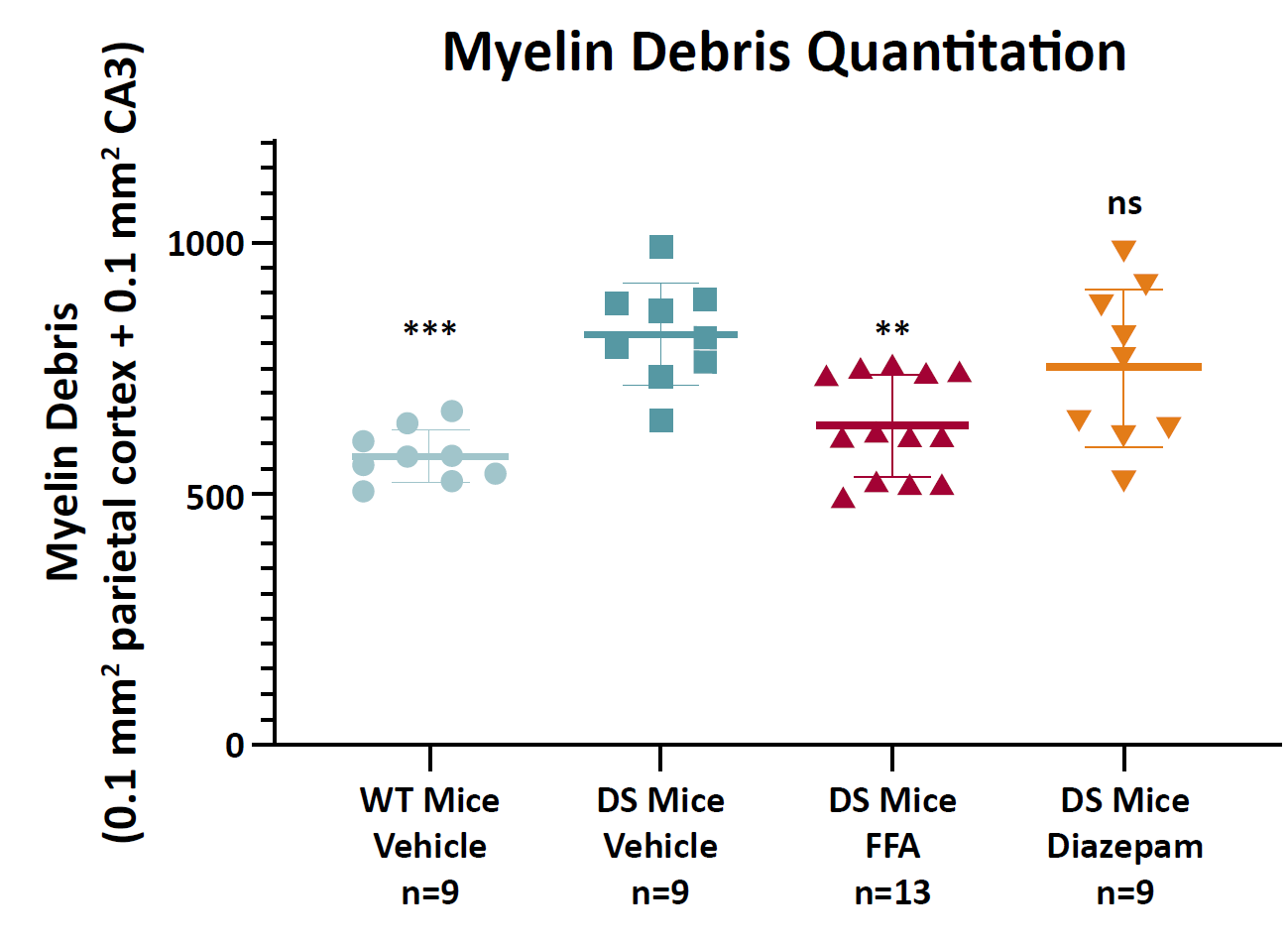


**Figure S3.** Activated microglia by CD11b+ immunostaining in corpus callosum (genu) and hippocampus (perforant pathway) in wild-type mice (n = 9) or surviving DS mice treated subcutaneously with vehicle (n = 9), 15 mg/kg/day fenfluramine (n = 13), or 10 mg/kg/day diazepam (n = 9) from PND 7 to PND 35-37. Representative images were taken of 5-µm sagittal brain sections immunostained with CD11b+ antibody for activated microglia. (A) Genu of the corpus callosum. (B) Perforant pathway of the hippocampus. Cyan (DAPI channel, CD11b) represents CD11b+ activated microglia. Purple overlays (Fast Red staining, Texas Red channel) provide neuroanatomical context. Scale bars: 60 µm using a 20X objective. DS, Dravet syndrome; PND, postnatal day; WT, wild-type.


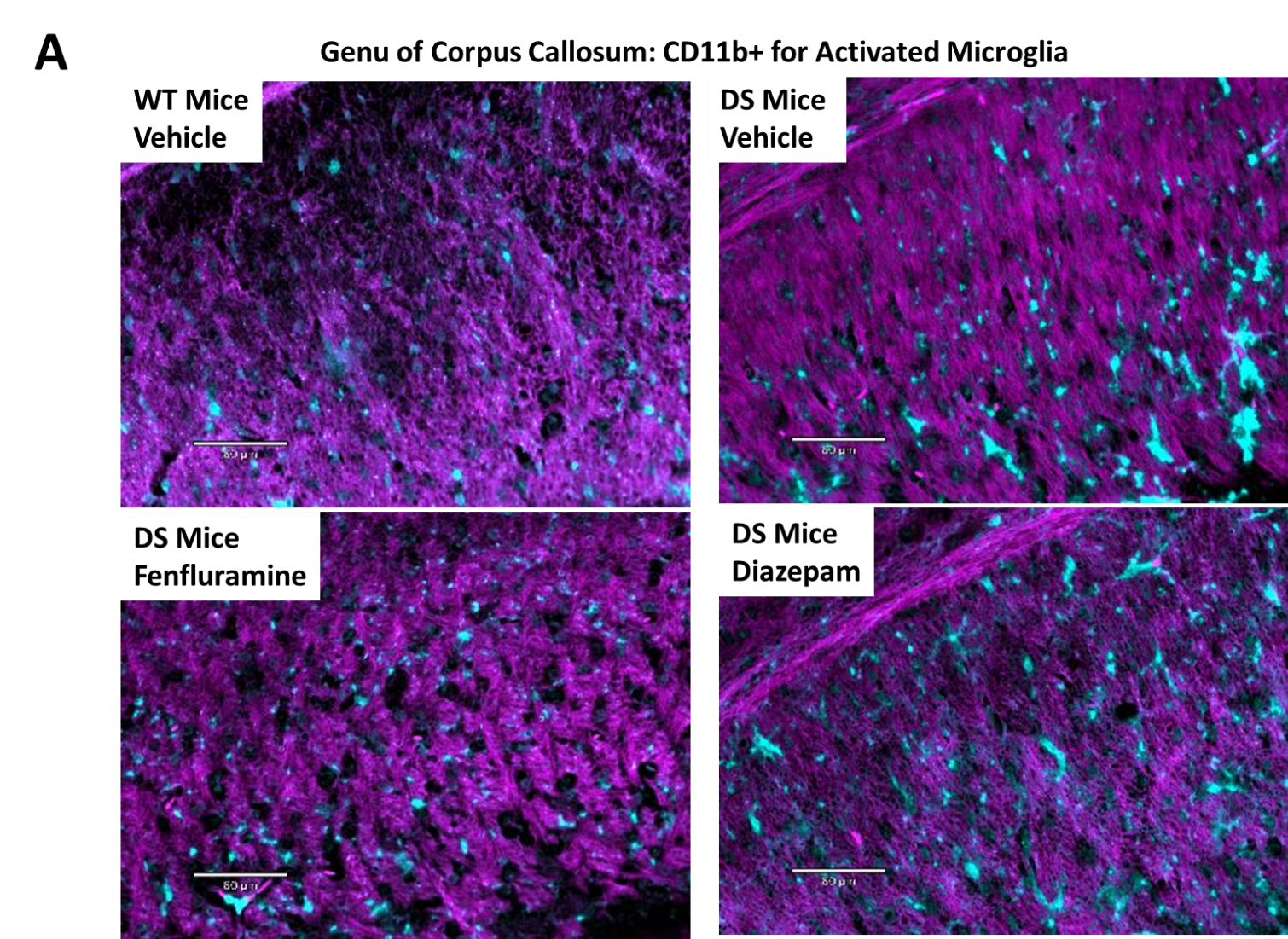


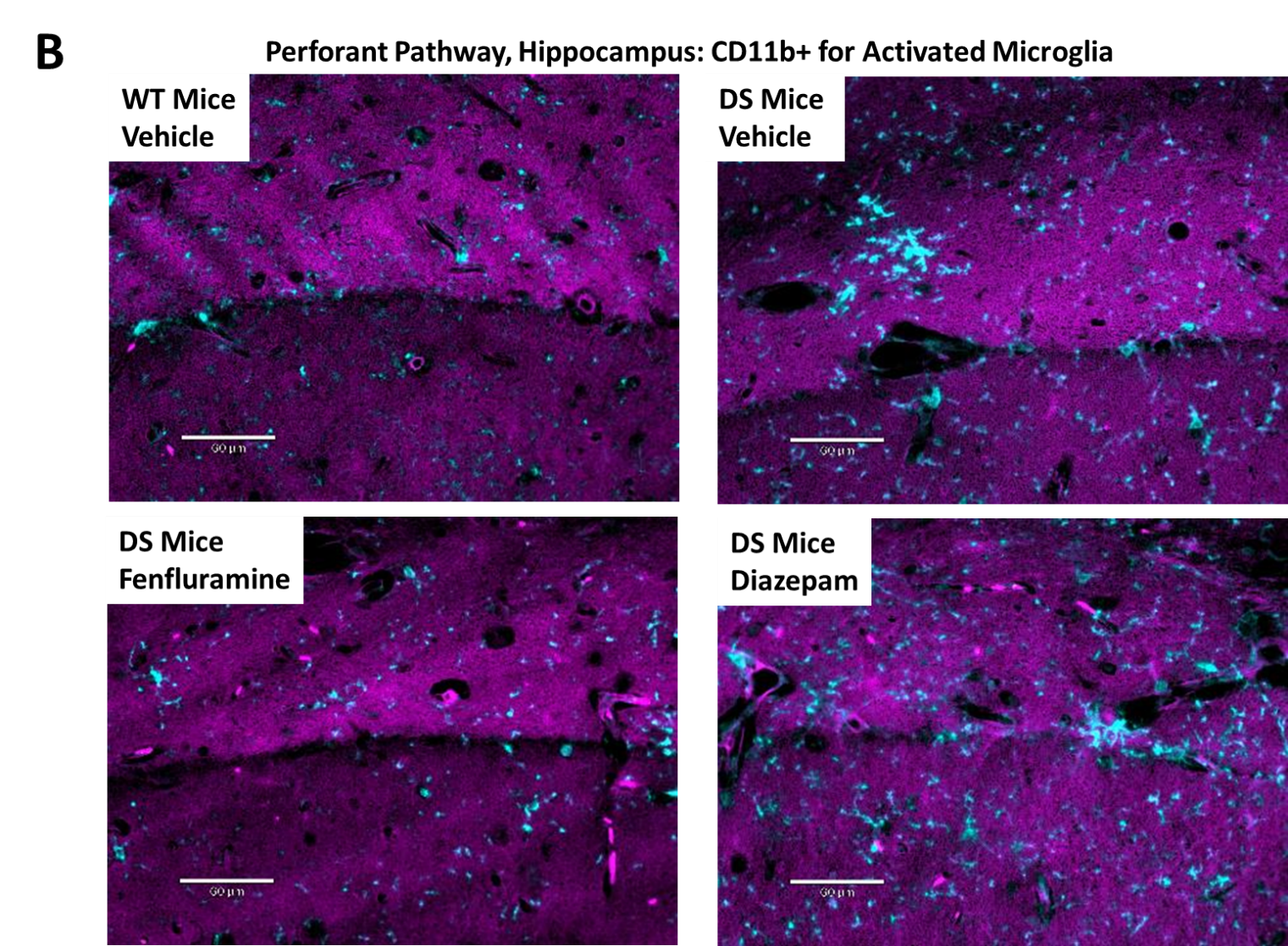


**Figure S4**. Image quantification of the effect of fenfluramine treatment on CD11b+ immunostaining of microglia in the hippocampus and corpus callosum. Images (one 5-µm sagittal section per mouse) were evaluated semi-quantitatively on a Likert scale of 0-5 (0, no activated microglia; 5, highly activated microglia). Data are plotted as mean ± SD; *P*-values were calculated by ANOVA with post-hoc Dunnett’s test for multiple comparison. ANOVA results: *P* = 0.0005. Dunnett’s multiple comparisons test results compared with DS vehicle-treated group: ** *P* < 0.01; * *P* ≤ 0.05; ns, *P* > 0.05 (left to right, vs DS Mice Vehicle: WT Mice Vehicle, *P* = 0.0013; DS mice FFA, *P* = 0.0284; DS Mice Diazepam, *P* > 0.9999). ANOVA, analysis of variance; DS mice, *Scn1a*^+/-^ Dravet syndrome mice; FFA, fenfluramine; WT, wild-type.


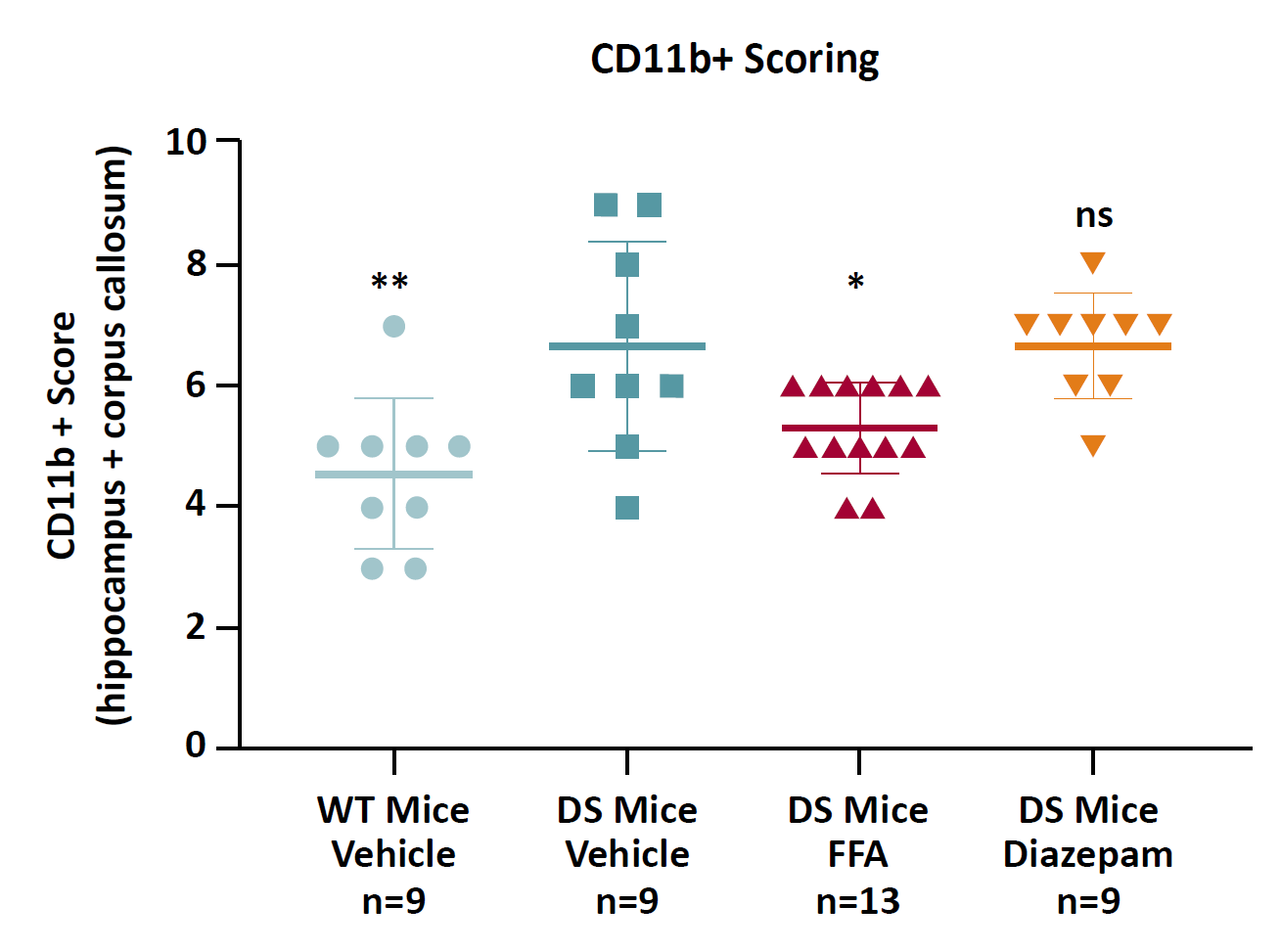


**Figure S5.** Apoptosis by TUNEL staining in 5-µm sagittal sections of the corpus callosum in wild-type mice or surviving DS mice treated subcutaneously once daily with vehicle (n = 9), 15 mg/kg fenfluramine (n = 13), or 10 mg/kg diazepam (n = 9) from PND 7 to PND 35-37. (A) Representative images from genu of the corpus callosum (scale bar, 130 µm; green [FITC] channel, 10X objective) and (B) manual image quantification of apoptotic nuclei by TUNEL using 4X magnification fields stitched together with Affinity Photo. In B, data are plotted as mean ± SD; *P*-values were calculated by ANOVA with post-hoc Dunnett’s test for multiple comparison. ANOVA results: *P* = 0.0054. Dunnett’s multiple comparisons test results compared with DS vehicle-treated group: ** *P* < 0.01 (left to right, vs DS Mice Vehicle: WT Mice Vehicle, *P* = 0.0061; DS mice FFA, *P* = 0.0733; DS Mice Diazepam, *P* = 0.0049). ANOVA, analysis of variance, DS mice, *Scn1a*^+/-^ Dravet syndrome mice; FFA, fenfluramine; PND, post-natal day; TUNEL, terminal deoxynucleotidyl transferase biotin-dUTP nick end labeling; WT, wild-type.


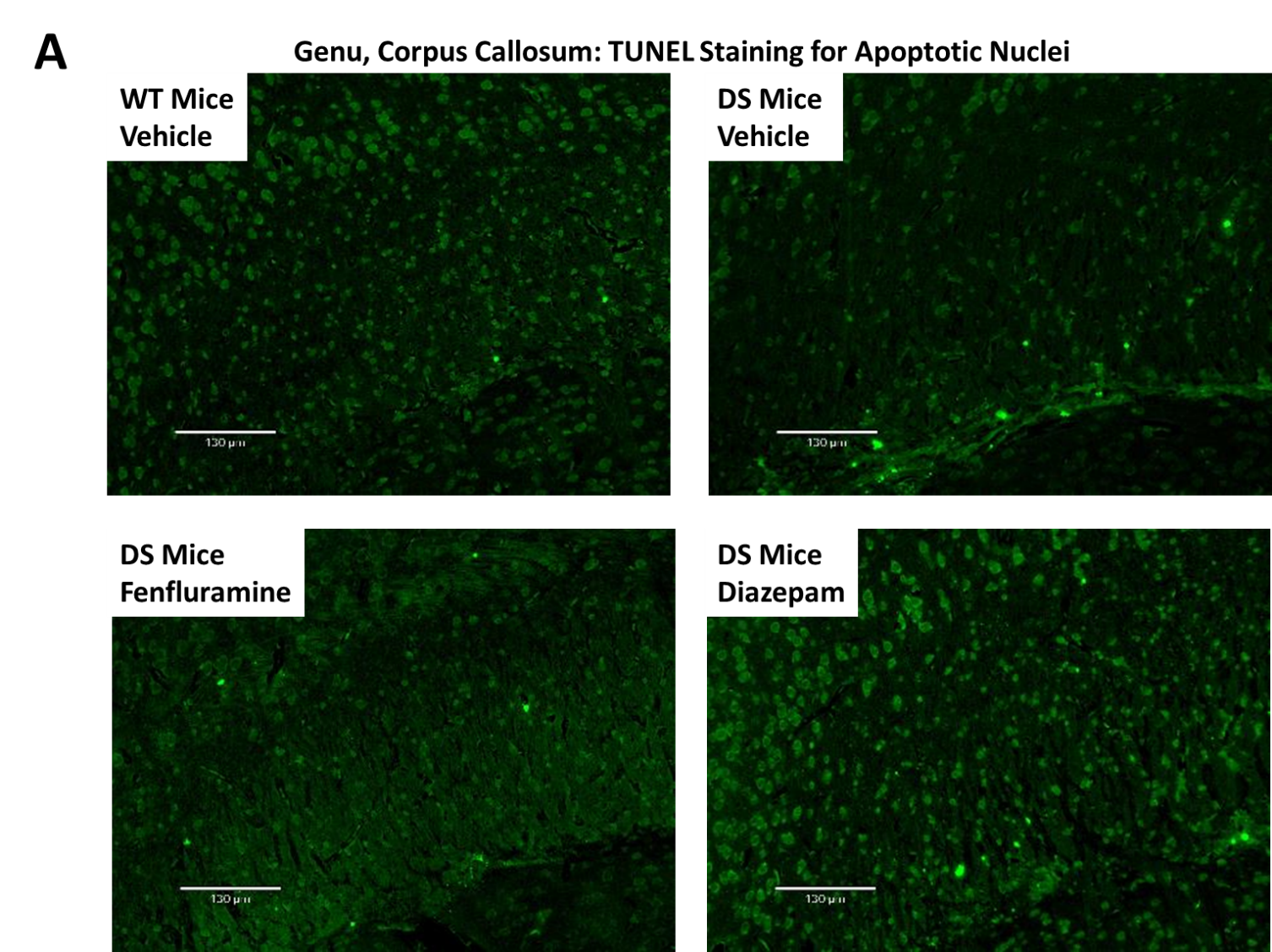


**B**

**Figure S6.** Effect of treatment on survival of *Scn1a*^+/-^ Dravet syndrome mice (DS mice). DS mice treated subcutaneously once daily with no treatment (n = 99), vehicle (n = 16), 15 mg/kg fenfluramine (n = 17), or diazepam (n = 24) from postnatal day (PND) 7 to 35-37. Survival was described using Kaplan-Meier methods using the Logrank Mantel-Cox test for significance (*P* = 0.0579 using a combined untreated/vehicle group [n = 115]; *P* = 0.1035 when the vehicle and untreated groups were independent; see inset).


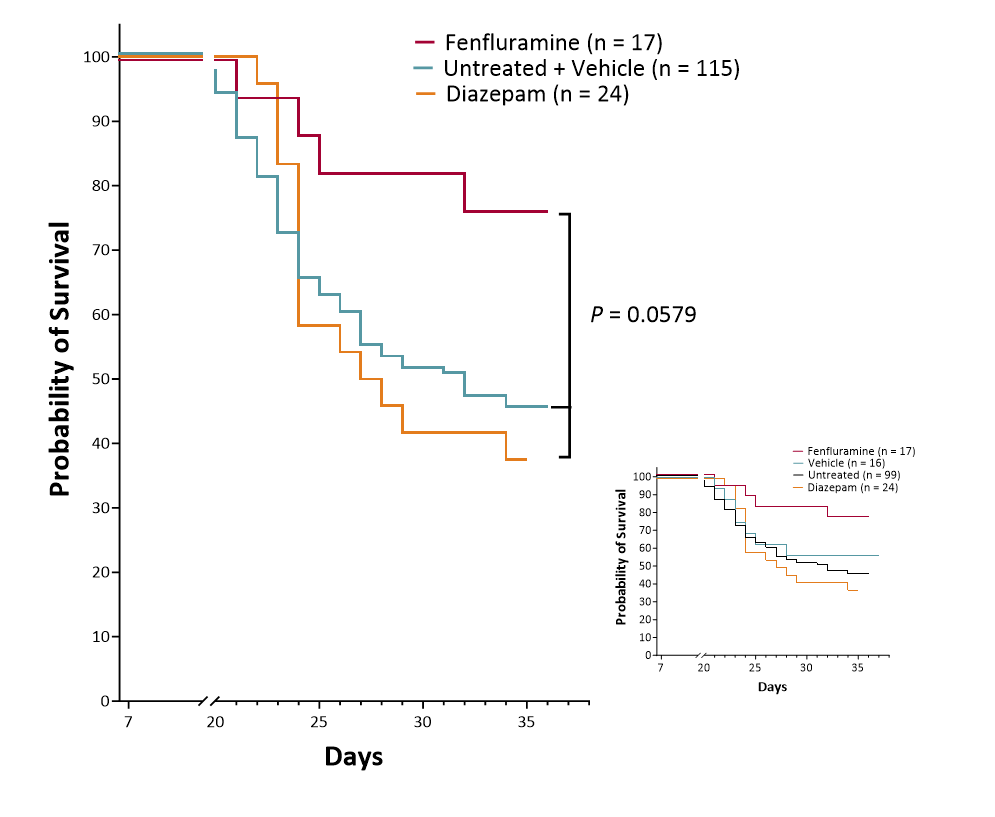


**Supporting Information: Videos (S1-3).** Video footage of 3 representative Dravet mice which died during the treatment period. Footage captures seizures prior to death. All 3 mice died immediately following a convulsive seizure rated 5 on the Racine seizure rating scale (*see* Racine RJ. Modification of seizure activity by electrical stimulation. II. Motor seizure. Electroencephalogr Clin Neurophysiol. 1972;32(3):281-94*.*).

**References:**

1. Tiraboschi E, Martina S, van der Ent W, Grzyb K, Gawel K, Cordero-Maldonado ML, et al. New insights into the early mechanisms of epileptogenesis in a zebrafish model of Dravet syndrome. Epilepsia*.* 2020;61(3):549-60.

2. McMullan J, Sasson C, Pancioli A, Silbergleit R. Midazolam versus diazepam for the treatment of status epilepticus in children and young adults: a meta-analysis. Acad Emerg Med*.* 2010;17(6):575-82.

3. Ochoa JG, Kilgo WA. The role of benzodiazepines in the treatment of epilepsy. Curr Treat Options Neurol*.* 2016;18(4):1-11.

4. Tanabe T, Awaya Y, Matsuishi T, Iyoda K, Nagai T, Kurihara M, et al. Management of and prophylaxis against status epilepticus in children with severe myoclonic epilepsy in infancy (SMEI; Dravet syndrome)--a nationwide questionnaire survey in Japan. Brain Dev*.* 2008;30(10):629-35.
